# Supplementary material for: Impact of Social Media Use on Mental Health within Adolescent and Student Populations during COVID-19 Pandemic: Review
Source: Int J Environ Res Public Health. 2023 Feb 15;20(4):3392. doi: 10.3390/ijerph20043392 (PMC9965407; doi:10.3390/ijerph20043392)
Supplement: Supplementary file 1 [file ijerph-20-03392-s001.zip › ijerph-2164632-supplementary.pdf]

Supplementary table S1: Search strategy used in PubMed and Web of Science Core Collection

Limiters/expanders: English; time: xx-30.04.2021.; Journal articles

DATABASE	#	Query	OUTPUT
PubMed	S1	(("covid 19"[All Fields] OR "covid 19"[Mesh Terms] OR "covid 19 vaccines"[All Fields] OR "covid 19 vaccines"[Mesh Terms] OR "covid 19 serotherapy"[All Fields] OR "covid 19 serotherapy"[Supplementary Concept] OR "covid 19 nucleic acid testing"[All Fields] OR "covid 19 nucleic acid testing"[MeSH Terms] OR "covid 19 serological testing"[All Fields] OR "covid 19 serological testing"[MeSH Terms] OR "covid 19 testing"[All Fields] OR "covid 19 testing"[MeSH Terms] OR "sars cov 2"[All Fields] OR "sars cov 2"[MeSH Terms] OR "severe acute respiratory syndrome coronavirus 2"[All Fields] OR "ncov"[All Fields] OR "2019 ncov"[All Fields] OR (("coronavirus"[MeSH Terms] OR "coronavirus"[All Fields] OR "cov"[All Fields]) AND 2019/11/01:3000/12/31[Date - Publication])) AND ("social media"[MeSH Terms] OR ("social"[All Fields] AND "media"[All Fields]) OR "social media"[All Fields] OR ("social networking"[MeSH Terms] OR ("social"[All Fields] AND "networking"[All Fields]) OR "social networking"[All Fields] OR ("social"[All Fields] AND "network"[All Fields]) OR "social network"[All Fields]) OR "facebook"[All Fields] OR ("twitter"[All Fields] OR "twitter s"[All Fields] OR "twitters"[All Fields]) OR ("youtube"[All Fields] OR "youtube s"[All Fields]) OR "WeChat"[All Fields] OR "Sina"[All Fields])) *Filters applied: English, from 1000/1/1 - 2021/04/30*	3,913
PubMed	S2	(("covid 19"[All Fields] OR "covid 19"[MeSH Terms] OR "covid 19 vaccines"[All Fields] OR "covid 19 vaccines"[MeSH Terms] OR "covid 19 serotherapy"[All Fields] OR "covid 19 serotherapy"[Supplementary Concept] OR "covid 19 nucleic acid testing"[All Fields] OR "covid 19 nucleic acid testing"[MeSH Terms] OR "covid 19 serological testing"[All Fields] OR "covid 19 serological testing"[MeSH Terms] OR "covid 19 testing"[All Fields] OR "covid 19 testing"[MeSH Terms] OR "sars cov 2"[All Fields] OR "sars cov 2"[MeSH Terms] OR "severe acute respiratory syndrome coronavirus 2"[All Fields] OR "ncov"[All Fields] OR "2019 ncov"[All Fields] OR (("coronavirus"[MeSH Terms] OR "coronavirus"[All Fields] OR "cov"[All Fields]) AND 2019/11/01:3000/12/31[Date - Publication])) AND ("social media"[MeSH Terms] OR ("social"[All Fields] AND "media"[All Fields]) OR "social media"[All Fields] OR ("social networking"[MeSH Terms] OR ("social"[All Fields] AND "networking"[All Fields]) OR "social networking"[All Fields] OR ("social"[All Fields] AND "network"[All Fields]) OR "social network"[All Fields]) OR "facebook"[All Fields] OR ("twitter"[All	**641**

		Fields] OR "twitter s"[All Fields] OR "twitters"[All Fields]) OR ("youtube"[All Fields] OR "youtube s"[All Fields]) OR "WeChat"[All Fields] OR "Sina"[All Fields]) AND (1000/01/01:2021/04/30[Date - Publication] AND "english"[Language]) AND ("mental health"[MeSH Terms] OR ("mental"[All Fields] AND "health"[All Fields]) OR "mental health"[All Fields]))<br><br>*Filters applied: English, from 1000/1/1 - 2021/04/30*	
Web of Science Core Collection	#1	ALL FIELDS: (mental health)<br><br>Indexes=SCI-EXPANDED, SSCI, A&HCI, CPCI-S, CPCI-SSH, ESCI, Timespan=All years	716,677
Web of Science Core Collection	#2	ALL FIELDS: (Social Media OR Social network OR Facebook OR Twitter OR Youtube OR WeChat OR Sina)<br><br>Indexes=SCI-EXPANDED, SSCI, A&HCI, CPCI-S, CPCI-SSH, ESCI Timespan= All years	459,570
Web of Science Core Collection	#3	ALL FIELDS: ("covid 19" OR "covid 19 vaccines" OR "covid 19 serotherapy" OR "covid 19 nucleic acid testing" OR "covid 19 serological testing" OR "covid 19 testing" OR "sars cov 2" OR "severe acute respiratory syndrome coronavirus 2" OR "ncov" OR "2019 ncov" OR "coronavirus" OR "cov")<br><br>Indexes=SCI-EXPANDED, SSCI, A&HCI, CPCI-S, CPCI-SSH, ESCI Timespan= All years	138,862
Web of Science Core Collection	#4	#1 AND #2 AND #3<br><br>Indexes=SCI-EXPANDED, SSCI, A&HCI, CPCI-S, CPCI-SSH, ESCI Timespan= All years	686
Web of Science Core Collection	#5	#3 AND #2 AND #1<br><br>Refined by: DOCUMENT TYPES: (ARTICLE) AND PUBLICATION YEARS: (2021 OR 2020)<br><br>Indexes=SCI-EXPANDED, SSCI, A&HCI, CPCI-S, CPCI-SSH, ESCI Timespan=All years	603
Web of Science Core Collection	#6	#3 AND #2 AND #1<br><br>Refined by: DOCUMENT TYPES: (ARTICLE) AND PUBLICATION YEARS: (2021 OR 2020) AND LANGUAGES: (ENGLISH)<br><br>Indexes=SCI-EXPANDED, SSCI, A&HCI, CPCI-S, CPCI-SSH, ESCI	587

Web of Science Core Collection	#7	#3 AND #2 AND #1<br><br>Refined by: DOCUMENT TYPES: (ARTICLE) AND PUBLICATION YEARS: (2021 OR 2020) AND LANGUAGES: (ENGLISH) AND WEB OF SCIENCE INDEX: (WOS.SSCI OR WOS.SCI-EXPANDED)	**495**
